# Improved techniques for measurement of nanolitre volumes of phloem exudate from aphid stylectomy

**DOI:** 10.1186/1746-4811-9-18

**Published:** 2013-06-17

**Authors:** Lachlan J Palmer, Lyndon T Palmer, Jeremy Pritchard, Robin D Graham, James CR Stangoulis

**Affiliations:** 1 School of Biological Science, Flinders University, 5042 Bedford Park, South Australia, Australia; 2 School of Agriculture Food and Wine, University of Adelaide, Waite Campus, 5064 Adelaide, South Australia, Australia; 3 School of Biosciences, University of Birmingham, Edgbaston, B152TT Birmingham, UK

**Keywords:** Aphid stylectomy, Exudate, Phloem, Volume measurement

## Abstract

**Background:**

When conducting aphid stylectomy, measuring accurate rates of phloem exudation is difficult because the volumes collected are in the nanolitre (nl) range. In a new method, exudate volume was calculated from optical measurement of droplet diameter as it forms on the tip of a severed aphid stylet. Evaporation was shown to decrease the accuracy of the measurement but was countered with the addition of water-saturated mineral oil. Volume measurements by optical estimation of the volume of a sphere suspended in oil was affected by the curvature of the oil surface. In contrast, measuring the exudate volume from optical measurement of droplet-diameter as formed on the tip of a severed aphid stylet, removes any inaccuracies due to oil surface curvature. A modified technique is proposed for measuring exudate volumes without oil by estimating the flow rate from photo-sequences of the collection period; a correction for evaporation is applied later.

**Results:**

A change in oil volume of ±1.75% from an optimum volume of 285 μl had a statistically significant effect on droplet measurement, under or over-estimating droplet volume due to optical effects caused by the oil surface. Using microscope image capture and measurement software, a modified method for measuring phloem volume in air was developed, by reducing air exposure during measurement to approximately 5 s for each measurement. Phloem volumes were measured using both techniques with measurements in air being on average 19.9 nl less (SD 18.87, p<0.001) than those made in oil, and there was a strong linear relationship (R^2^=0.942) between the techniques. This linear relationship enabled the development of a correction equation with no significant difference at the 5% level between corrected volumes and actual volumes measured under oil.

**Conclusions:**

This study showed that oil has a significant role in countering evaporation but oil volume must be carefully optimised for optical measurement of droplets to ensure measurement accuracy. A linear correction factor was generated to correct the volumes measured in air for loss due to evaporation and the method provides for a much simpler alternative to previous approaches for measuring exudation rates and volumes from a cut aphid stylet.

## Background

Since the first report on collecting phloem exudate after insect stylectomy [1], the technique has been used to gain access to the phloem for the study of a broad variety of its physiological, mechanical and molecular properties (see reviews [2-4]). Owing to the small exudate volumes flowing from severed stylets, evaporation has been identified as a significant factor in accurate quantification of phloem exudate components. In a review of the work done on the *Salix* sp.-*Tuberolachnus salignus* model [3], evaporation of phloem exudate was identified as a significant concern for quantitative analyses. At that time there was no system available for experimentally countering evaporation in this model. With the development and use of laser stylectomy [5] and radio frequency microcautery stylectomy [6], the number of potential insect-host models was expanded to 46 plant species [7]. The issue of evaporation was still of concern and has been demonstrated to cause increases in osmotic pressure measurements [8,9], with as much as a two-fold increase in osmotic pressure being reported for droplets collected in air when compared to those collected in oil [9]. Accordingly, various approaches were developed to counter this problem. The main technique involved minimising evaporation through the use of mineral oil, where a droplet of oil is placed directly onto the plant surface, over the severed stylet [6]. Containers have been deployed to surround stem sections that are then filled with oil after stylectomy [9]; another technique of gluing wells, to contain oil, onto the plant surface prior to stylectomy [10] or after stylectomy [11] is commonly used. Other techniques used for minimising evaporation have included placing a capillary directly over the severed stylets of aphids [1] or plant hoppers [12] and collecting from a severed stylet into an oil back-filled capillary and adjusting for evaporation losses [13].

Accurate and precise measurements of exudate volume are difficult. Phloem volumes ranging from as little as 2.1 nl [13] to as much as 100 μl [14] have been reported in individual collections from a single stylet. Volume measurements have been made in a variety of ways, including direct measurement from bulked collections; averaging across a number of stylets [10]; or combining the length of collection with the time taken for a droplet to reach a certain diameter under oil [11] or in air [13]; the size of the collection droplet suspended in oil [15]; or the length of liquid within a micro-capillary of a known diameter [12]. Recently it has been noted that oil can cause interference in the analysis using capillary electrophoresis with laser-induced fluorescence detection [13]. It has also been the experience of the authors that paraffin oil can seriously interfere with metabolite profiling of phloem samples by GC-MS, by affecting the baseline resolution and by contaminating the mass spectrum response for low abundance compounds (unpublished data). It is the author’s concern also that for measurements made under oil, the shape of the oil surface may impact significantly on volume measurements made within the oil. The convex surface of the meniscus formed on the surface of an oil droplet or by overfilling a well with oil will have a positive focal length and if the droplet is in a position less than two times the focal length behind the surface, it will be magnified [16]. In the case of a convex meniscus formed by under filling a well, the focal length will be negative and the droplet will appear reduced in size regardless of its position [16]. The aim of this work was to explore the effect that oil surface shape, due to oil volume, has on optical measurements of droplets contained within the oil. It also details a refinement to measuring droplets in air by making use of image capture and measurement software to shorten exposure times to air and to allow a measurement using a single time length for all measurements in air.

## Results

### Establishing optimum oil volume

**Figure 1:**
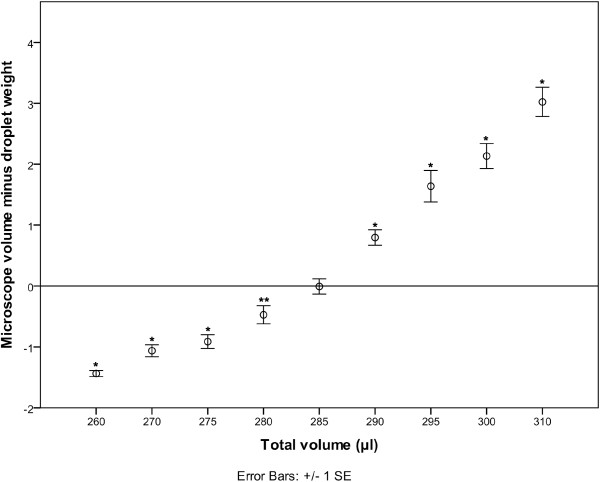
plot of the difference between the mean droplet volume measured optically (μl) and by weight (mg) for each total liquid volume (oil+MilliQ water) with significant differences indicated (*=p>0.001, **=p>0.01).

The cap from a 1.5 ml micro-centrifuge tube was selected to contain the oil for volume measurement owing to its hydrophobic surface which enabled droplets of aqueous solutions to remain spherical. Nine oil volumes were tested using 10 μl droplets of MilliQ water. Figure 1 shows the difference between the average microscope measurement and weight measurement for the droplets at each total volume (oil+water). Clearly the shape of the liquid surface is having an impact on droplet measurement using microscope measurement. There was no significant difference in droplet weights across all total volumes (p = 0.123), but the total volume had a significant effect on the microscope measured volume (p < 0.001). For a total volume of 285 μl there was no significant difference between the volume measured by weight and that measured using the microscope technique (p=0.954). For all other total volumes there were statistically significant differences (see Table 1 for a summary of the mean differences). From these results it can also be seen that a total volume change of 1.75% or 5 μl is enough to produce a statistically significant difference in the microscope measurement of the droplet. Based on these results a volume of 285 μl oil was selected for measuring nl droplets. Inductively Coupled Plasma Mass Spectroscopy (ICP-MS) analysis of nl droplets of a 1000 mg kg^-1^ cobalt (Co) stock solution was used to confirm 285 μl of oil as a suitable volume for the measurement of nl volumes. Co was selected as it is a single mass element with high sensitivity and reduced risk of contamination owing to its very low abundance in plant tissues. Attempts were made to repeat the oil volume trial with nL droplets of Co stock solution, but results were much more variable. This is most likely due to differences in liquid surface curvature when oil volumes are sub-optimal. Small droplet size could also contribute to the variability as the larger volume of a 10 μl Milli-Q droplet showed less variability but nL volume of droplets of Co solution still indicated that 285 μl was closest to the ideal volume.

**Table 1:** Percent error between droplets measured by weight and optically for different total liquid volumes (oil+MilliQ volume) and the respective percent difference in oil volume when compared to the selected optimal total volume of 285 μl

**Total liquid volume (μl)**	**n**	**% error**	**% difference in oil volume from optimum volume**
260	10	−14.4	8.77
270	10	−10.8	5.26
275	10	−9.2	3.51
280	14	−4.9	1.75
285	15	−0.1	0
290	15	8.1	1.75
295	13	17.0	3.51
300	15	21.6	5.26
310	10	31.1	8.77

(%error=mean optical volume – mean droplet weight * 100/ mean droplet weight).

### Standard addition

Owing to the analysis of Co by ICP-MS it was necessary to test if the presence of oil would have an effect on the nebulizer or plasma performance (matrix effect). This was tested using a standard addition trial. For the results used in the standard addition test there was a significant difference between the results with and without an internal standard (ISTD) correction. The un-corrected (no ISTD) data were on average 0.18±0.05 μg L^-1^ (n=19, p = 0.001) higher than the ISTD corrected measurements. This is equivalent to an average reduction of 1.5%±0.039 (n =19) in sample concentrations when results are corrected using the ISTD. Therefore standard additions were calculated using both data sets.

There was no significant difference between the values estimated from the standard addition samples measured with and without ISTD correction (n=3, p=0.096). For the standard addition results calculated using values without ISTD correction there was no significant difference between the estimated values and the original sample measurements made with and without ISTD correction (n = 3, p = 0.144 and 0.979 respectively). For the standard addition results calculated with ISTD corrected values the estimated values were significantly different from the original results for samples measured with and without ISTD correction. The standard addition estimate using ISTD corrected values was 0.37±0.05 μg L^-1^ (p=0.018) higher than the ISTD corrected values and 0.25±0.03 μg L^-1^ (p=0.018) higher than the values without ISTD correction.

This result indicates that there is no matrix effect caused by the oil in the analysis by ICP-MS. It also indicates that the use of the internal standard is not required and that the use of the ISTD may actually be producing an artificial matrix effect. Therefore for the remaining work, results from ICP-MS analysis were calculated without ISTD correction.

### Method for measuring nl volumes

Droplets (n=28) of 1000 mg L^-1^ Co stock solution were measured in 285 μl of oil using the microscope and by ICP-MS. Microscope-measured volumes ranged from 86 nl to 194 nl (mean 111.8 nl, SD 23.0). There was no significant difference between the microscope measurement and ICP-MS volume measurement (p=0.799). On average, the microscope measurement underestimated the volume by 0.15 nl, SD 3.02.

### Oil vs Air

To test the oil measurement technique on real phloem exudate collections and to determine the accuracy of volumes measured based on an estimated flow rate made from droplets measured in air, samples were collected from 71 successfully severed stylets. Each collection was measured using both methods and the estimated volumes compared.

**Figure 2:**
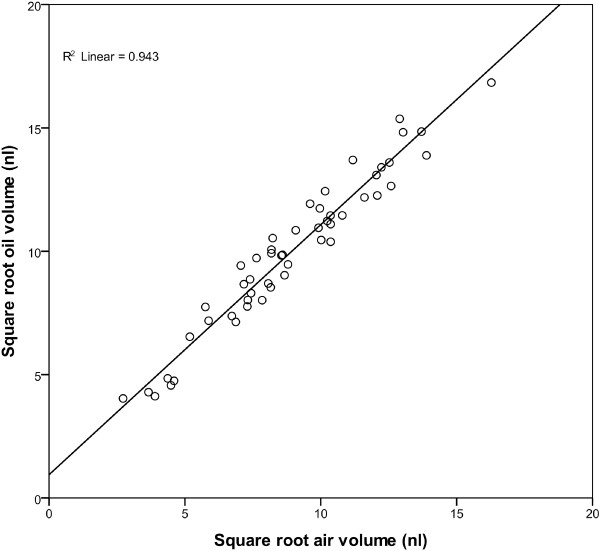
Linear regression plot of square root transformed phloem collection volumes measured in 285 μl of oil versus square root transformed volumes estimated using flow rates measured in air and collection length for the 51 samples in the Cal set.

The average collection time was 45.6 min, SD 7.21. Flow rates measured in air averaged 1.8 nl min^-1^, SD 1.3 with a range of between 0.07 nl min^-1^ to 5.9 nl min^-1^. The average estimated collection volume in air was 83.4 nl, SD 56.6 with a range between 2.0 nl and 269.2 nl. The average oil measured volume collected was 103.3 nl, SD 63.8 with a range of between 2.89 nl and 283.5 nl. There was a significant difference in volume measured using the two techniques, with the volume measured under oil being on average 19.9 nl larger (SD 18.9, p < 0.001) than that of the volume measured in air. The volumes of the 71 phloem collections measured using both techniques were transformed by taking the square root to ensure data was normally distributed. From the 71 measured samples, a random subsample of 51 collections (Calibration (Cal) set) was selected and used to produce a linear regression and correction equation. The remaining 20 samples were used for validation of the method (Validation (Val)set). For the Cal set subsample, there is a significant regression between the two techniques (R^2^=0.942, p  >0.001) (Figure 2). From the regression analysis, the following equation can be calculated for correcting measurements made in air:

$$\mathrm{Corrected}\;\mathrm{volume}\;=\;{\left(\sqrt{\mathrm{Air}\;\mathrm{vol}},,\;,\times ,\;1.014\;+\;0.942\right)}^{2}$$

This equation was applied to volumes measured under air from the Val set to calculate the corrected volume for these samples. There was no significant difference between the corrected volume and the volume measured in oil for the Val set (p = 0.932). For the samples in the Val set, on average the corrected air measurement was 0.5 nl SD 23.3 larger than the direct measurement in air.

## Discussion

The use of oil in the collection of phloem exudate had been used primarily to reduce errors caused by evaporation. Evaporation during collection in air has been shown to cause a significant increase in magnitude and variance of measurements of osmotic pressure in phloem exudate when compared to collections made under oil [8,9]. The use of oil to minimise evaporation during the collection of phloem exudate by insect stylectomy is the accepted method for reducing evaporation effects [17]. Moreover the results from this work have demonstrated that the shape of the oil surface plays a significant role in accurate measurement when using optical volumetric measurement techniques. Methods making use of a droplet of oil either on the plant surface [6] or for measurement of complete collection [15] may lead to an overestimation due to the convex surface of a droplet. In this study, at the largest oil volume there was an average error of 31.2% when compared to droplet weights (see Table 1), so droplets measured in droplets of oil on a flat surface may potentially over-estimate by as much as 30%; a significant potential source of variability for quantitative analysis. For techniques using wells located on the plant surface [11] or surrounding the plant [9] it may be possible to standardise the volume of oil used to ensure correct measurements. In these published methods there is no mention of the well/container size so it is difficult to know what volumes are involved but any inconsistency in the size of the well or oil container may affect oil volume and thus create errors in volume measurements. In Table 1 it can be seen that a variation in oil volume of less than 2% has a significant effect on the accuracy of volume measurements under oil. So it can clearly be seen that small variations in oil volume will significantly affect volume measurement.

For analytical techniques where oil may cause interferences, collection and measurement in air has been used with a correction factor [13]. The correction factor developed by Gattolin et al. was a power function due to the method used where the time taken for the droplet to reach a specific size is used to calculate flow rate. Slower-flowing stylets require longer measurement times and so are exposed to greater evaporation bringing about the power function relationship. This work has demonstrated that image capture and software can be used to reduce the time that droplets are exposed to air and when combined with the improved accuracy of the oil measurement, a linear correction equation can be calculated. Both techniques could be used in future work exploring the elemental, metabolite and protein composition of phloem exudate collected from single stylets. This will help expand on the current body of work in phloem proteomics [10,18], micronutrient transport [19] and phloem metabolomics [20].

## Conclusion

Oil is commonly used to counter evaporation during the collection of phloem by aphid stylectomy. This work demonstrates that oil volume has a significant effect on the accuracy of optically measured volume. When oil volume is optimised, optical measurement of nl volumes in oil is extremely accurate. Oil also has the potential to interfere with analysis of phloem exudate and this work demonstrates an improved technique for measuring phloem volume in air. An equation to correct for evaporation was able to be calculated to ensure the accuracy of volume measurement was maintained.

## Methods

### Solutions

Oil (Paraffin oil, LABCHEM™, Ajax Finechem) was produced by mixing two parts oil with one part Milli-Q™ water and shaking for 2 hours on a rotary shaker, then allowed to separate after which excess water was removed. All nitric acid solutions used in this research were prepared using Instrument Quality acid (Seastar) diluted v/v in Millipore Milli-Q™ water (>18.2 MΩ cm^-1^) using acid washed volumetric glassware. All ICP-MS standards used in this research were prepared using a Gilson dual syringe auto-dilutor from certified single element stock solutions (1000 μg ml^-1^ High-Purity Standards). All final solutions contain 2 μg L^-1^ Indium (In) as an ISTD to aid spectrometric analysis.

### Volume measurement under oil

Using a positive displacement pipette, volumes of oil were measured onto the lid removed from a 1.5 ml centrifuge tube. For μL volume droplets, droplets were deposited onto the oil surface using an air displacement pipette and weighed to the nearest 0.1 mg. For nL volume droplets, a glass microcapillary was used to deposit the droplet immediately beneath the oil surface. All droplets were photographed in oil using a Leica microscpe (M165C or MZ16) with an attached camera (DFC295 or DFC280). The radius (μm) of the droplet was measured using the Leica Application Suite (v 3.6.0) with the additional Interactive Measurements Module and the volume of the droplet estimated.

### Using ICP-MS to measure nL volumes

After samples were photographed, a 1.5 ml centrifuge tube was placed over the cap containing the oil and droplet and centrifuged briefly at 13,000 rpm. 1 ml of diluting solution (2% nitric acid , 2 μg L^-1^ In) was added, vortexed briefly, inverted 10 times and poured into a 15 ml Greiner tube. This was repeated with four 1 ml rinses of the dilution solution. For the final rinse, a pipette was used to remove the rinse solution. A final 5 ml of diluting solution was added to the 15 ml Greiner tube to make to 10 ml+oil. 3 ml aliquots were loaded into 6 ml polypropylene vials (G3160-65303, Agilent) and analysed by ICP-MS (Agilent 7500cx with integrated autosampler). A 0.1 ml min^-1^ MicroMist nebulizer (Glass Expansion) was used with 0.89 mm ID peristaltic tubing (0.89-ORG, Glass Expansion). The reaction cell was run in Helium mode with a gas flow rate of 4.5 ml min^-1^ and the equipment was tuned using the autotune function. Mass spectrum data acquisition was repeated three times per sample. For each replication three points were used for integration, for Cobalt (Co), mass 59 was integrated for 3 seconds per point and for In, masses 115 and 118 were integrated for 1 second per point. Fe masses 56 and 57 and Zn mass 66 were also acquired at 1 second per point, to check for contamination (data not shown). This gave a total acquisition time of 76.38 seconds per sample.

### Standard addition

From the nl samples, three Co and one blank sample were selected. After the samples had been diluted, four 1.5 ml aliquots were taken. To each aliquot from each sample, 1.5 ml of a calibration standard (0, 5, 10 and 20 μg L^-1^) were added, mixed well and the 3 ml volume run as detailed above.

### Plant material

Wheat (*Triticum aestivum* L. genotype ‘Samnyt 16’) seedlings were grown in 70x100 mm pots in Debco™ Green Wizard potting mix within a growth room. Growth room conditions were 13/11 h light/dark at 20°C/10°C with a minimum of 400 μmol m^-2^ s^-1^ light at the leaf surface. Plants were transferred to a glasshouse immediately prior to addition of aphids. Plants were grown until full emergence of the inflorescence and used for up to approximately 20 days after anthesis.

### Aphid stylectomy

Aphid stylectomy procedures were adapted from Downing and Unwin (1977). Aphids were secured to wheat plants using specially prepared cages; a minimum of 12 h prior to stylectomy. Aphids were taken from an anholocyclic *Sitobion miscanthi* (Indian grain aphid) culture maintained at Flinders University on wheat plants kept under glasshouse conditions. Only apterous aphids were used in the experiments. Plants were watered to saturation at time of the addition of aphids. On the day of collection, plants were taken from the glass house between 2 and 3 pm (Australian central daylight savings time, ACDT) and stylets cut up until 5 pm ACDT. Stylectomy was performed using high-frequency micro-cauterisation under a Leica microscope (M165 C or MZ16) using an electrolytically-sharpened tungsten needle in combination with a micromanipulator. Exudate samples were collected using oil backfilled glass micro-capillaries (30–0017, Harvard Apparatus) pulled using a capillary puller (Narishige). Collections were made between February and April with the relative humidity during collection ranging from 41% to 50%.

### Microscope measurement of nL phloem exudate collections

Measurement of exudate collections was modified from previously reported methods [11,13]. The volumes for each collection were measured twice by multiplying an estimated average flow rate by the length of collection and total volume of the finished collection measured in oil contained within a 1.5 ml centrifuge tube cap (as detailed previously). Exudate flow rates were estimated from photo sequences taken using a Leica microscope (M165C or MZ16) with an attached camera (DFC295 or DFC280) and the multi-time module from the Leica Application Suite software (v3.6.0). Photo sequences were taken immediately after obtaining an exuding stylet, approximately every 15 minutes during collection and immediately prior to the end of the collection. Photo sequences consisted of five photos with a one second interval between each photo. The droplet radius for each photo in a sequence was measured using the interactive measurement module within the Leica Application Suite and an estimate of droplet volume calculated. Using the time interval between each photo in a sequence the exudation flow rate was estimated from the change in volume between photos. The average of the estimated flow rate from all sequences in each collection was multiplied by the respective collection length to give an estimate of the collection volume.

## Abbreviations

Oil: Water saturated mineral oil; ICP-MS: Inductively coupled plasma-mass spectrometry; ISTD: Internal Standard; ACDT: Australian central daylight savings time

## Competing interests

The authors declare that they have no competing interests.

## Authors’ contributions

LJP helped in conceiving study, collected phloem samples, did ICP-MS analysis, statistical analysis and drafted the manuscript. LTP was involved ICP-MS analysis and study design. JP provided the stylectomy collection methods and assisted in study design. RG helped in the design of the study and was responsible for the idea of using the ICP-MS for measuring droplet volume. JS participated in conception and design of the study and assisted with drafting of manuscript. All authors read and approved the final manuscript.
